# Current Therapeutic Strategies and Novel Approaches in Osteosarcoma

**DOI:** 10.3390/cancers5020591

**Published:** 2013-05-24

**Authors:** Kosei Ando, Marie-Françoise Heymann, Verena Stresing, Kanji Mori, Françoise Rédini, Dominique Heymann

**Affiliations:** 1 INSERM, UMR 957, 1 Rue Gaston Veil, 44035 Nantes, France; E-Mails: mfheymann@chu-nantes.fr (M.-F.H.); verena.stresing@univ-nantes.fr (V.S.); francoise.redini@univ-nantes.fr (F.R.); dominique.heymann@univ-nantes.fr (D.H.); 2 Physiopathologie de la Résorption Osseuse et Therapie des Tumeurs Osseuses Primitives, Université de Nantes, Nantes Atlantique Universités, 1 Rue Gaston Veil, 44035 Nantes, France;; 3 Equipe Labellisee Ligue 2012, Nantes, 44035 France; 4 Nantes University Hospital, Nantes 44035, France; 5 Department of Orthopaedic Surgery, Shiga University of Medical Science, Tsukinowa-cho, Seta, Otsu, Shiga 520-2192, Japan; E-Mail: kanchi@belle.shiga-med.ac.jp

**Keywords:** osteosarcoma, chemotherapy, surgery, radiotherapy, multidisciplinary treatment, emerging drugs

## Abstract

Osteosarcoma is the most frequent malignant primary bone tumor and a main cause of cancer-related death in children and adolescents. Although long-term survival in localized osteosarcoma has improved to about 60% during the 1960s and 1970s, long-term survival in both localized and metastatic osteosarcoma has stagnated in the past several decades. Thus, current conventional therapy consists of multi-agent chemotherapy, surgery and radiation, which is not fully adequate for osteosarcoma treatment. Innovative drugs and approaches are needed to further improve outcome in osteosarcoma patients. This review describes the current management of osteosarcoma as well as potential new therapies.

## 1. Introduction

Osteosarcoma, the most common primary malignant bone tumor, usually arises in the metaphysis of long bones such as the distal femur, proximal tibia, and proximal humerus during the second decade of life. A second smaller peak occurs between 60 to 80 years [1,2]. Overall, osteosarcoma has a moderate incidence rate, with 10 to 26 per million new cases worldwide each year [3]. 

**Figure 1:**
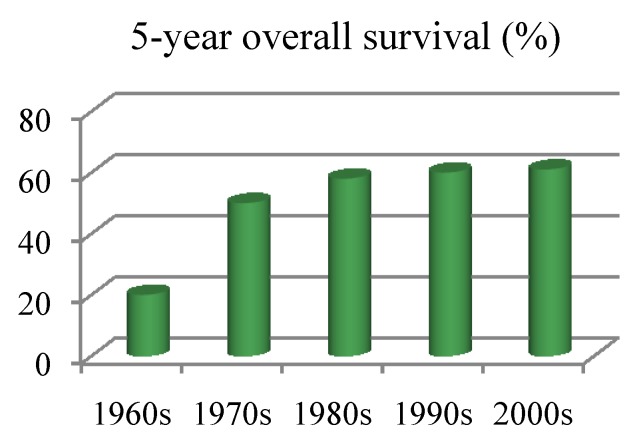
Five year overall survival of localized osteosarcoma (from Allison *et al*. [9]).

The 5-year survival in osteosarcoma in the first half of the 20th century was less than 20% [4]. These patients were mainly treated by limb amputation and most of them died of lung metastases [5]. Since then, long-term survival for patients with localized osteosarcoma has improved to approximately 60% due to the newly-introduced multi-agent chemotherapy together with gradually-improved surgical techniques in the 1970s (Figure 1), but has remained largely unchanged since then (Figure 1). By contrast, the long-term survival of patients with metastatic osteosarcoma still remains at 25–30% [6,7,8]. In fact, the main chemotherapeutic agents used for the treatment of the metastatic disease have not changed much over the course of the past 30 years [9]. The present review focuses on summarizing current strategies for osteosarcoma treatment and gives an overview of novel therapeutic developments in the field of bone sarcomas [10].

## 2. Current Therapies

Therapeutic approaches are generally based on various factors: tumor entity, tumor stage, age, gender, general condition, quality of life, life expectancy, *etc.* Today, there are three major therapeutic options for patients suffering from osteosarcoma: surgery, chemotherapy, and palliative radiotherapy. The standard treatment associates both neoadjuvant and adjuvant chemotherapies and surgical treatment. Radiotherapy is administered with a surgical resection in some cases.

### 2.1. Surgical Treatment

**C**urrently, surgery remains an indispensable part of osteosarcoma treatment together with chemotherapy. Jaffe *et al*. reported that only 10% of patients with osteosarcoma were cured exclusively with chemotherapy and highlighted the significance of surgery in osteosarcoma treatment [11]. The aim of surgery must be a complete tumor removal with a wide margin of normal tissue in order to avoid local recurrence and improve overall survival. Today, the extent of surgical resection and its optimal margin is determined according to Enneking’s tumor staging (Table 1). The surgical treatment of osteosarcoma consists of several options such as amputation, limb-salvage with endoprosthetic or biological reconstruction, rotationplasty, *etc*. The choice of these options depends on tumor grade, location, and response to neoadjuvant chemotherapy [12]. However, in spite of the advanced surgical techniques in the modern era, the local recurrence rate in patients with non-metastatic osteosarcoma has been reported as high as 46% [13]. Thus, surgical procedures should be conducted together with conventional chemotherapy for both local tumor and micro-metastases control [14,15]. 

Taking into account the various factors including type, grade, and location of tumor, tumor response to chemotherapy, functional outcome and patients’ preference, surgeons are required to determine the optimal surgical procedure for patients with osteosarcoma. The family of the patient should also positively participate in this decision making process [16]. 

**Table 1:** Enneking’s surgical staging system of bone sarcoma [17].

Stage	Site	Grade	Metastasis
**IA**	Intracompartmental	Low	No
**IB**	Extracompartmental	Low	No
**IIA**	Intracompartmental	High	No
**IIB**	Extracompartmental	High	No
**III**	Any	Any	Regional or distant

#### 2.1.1. Amputation

Amputation was the main and standard therapeutic option for patients with osteosarcoma before the 1970s, and the 5-year overall survival rate with amputation alone was 5–23% [18,19]. Today, amputation is not a first choice anymore owing to advances made in chemotherapy, surgical techniques, surgical devices, and diagnostic methods. In fact, approximately 90% of patients currently undergo wide resection with limb-sparing surgery [20,21]. There is no difference in survival rate between patients undergoing limb-salvage procedures and amputations, whereas there is a high trend of local recurrence with limb-salvage [16,21,22]. If the tumor cannot be removed with safe margins, amputation should be taken into account as another surgical option [23] (Table 2). 

**Table 2:** Enneking’s criteria for surgical procedures [17].

Margin	Dissection	Surgical option	
		Limb-salvage	Amputation
**Intralesional**	Within tumor, intracapsular	Intracapsular, piece meal	Intracapsular
**Marginal**	Within reactive zone, extracapsular	Marginal en bloc	Marginal
**Wide**	Beyond reactive zone; Intracompartmental through normal tissue	Wide en bloc	Wide through-bone
**Radical**	Extracompartmental, normal tissue	Radical en bloc	Radical extra-articular

Several complications are caused by amputation: wound necrosis, infection, overgrowth of bone in children, neuroma, stump pain, and phantom limb pain [16,24]. In particular phantom limb pain, the sensation that the amputated extremity is still present with burning and cramping pain, which is specific to amputations, has been reported [24]. This symptom can be induced by multiple factors: pre-amputation pain, age, chemotherapy in bone tumors, *etc.* [25,26].

#### 2.1.2. Limb-Salvage Surgery

Limb-salvage surgery is currently the gold standard in osteosarcoma treatment. The aim of limb-salvage surgery is to maximally preserve a limb with a satisfactory function and to avoid the psychological and cosmetic problems caused by amputations [27]. Recent advances in chemotherapy, surgery and diagnosis have enabled a major shift from amputation to limb-salvage surgery in tumor resection [28,29,30]. Furthermore, there is no difference in disease-free survival between limb-salvage surgery and amputation in patients with high-grade osteosarcoma [21,23]. However, it is important to keep in mind that limb-salvage surgery carries a higher risk of local recurrence than amputation [21,23]. Thus, limb-reconstruction should be considered carefully. The tumor mass should be completely resected including the reactive zone [31]. At present, surgeons determine the resection margins, which are defined as intralesional, marginal, wide, and radical, according to the Enneking’s criteria (Table 2) [32,33]. These margins are associated with the local recurrence risk together with the efficacy of chemotherapy [23,34]. In summary, limb-salvage surgery should be recommended under conditions that the safe surgical margins can be achieved and that this surgical treatment will be performed by experienced surgeons [13].

#### 2.1.3. Limb Reconstruction

The options available for reconstruction after limb-salvage tumor resection include endoprosthetic replacement, allografts, autografts, and rotationplasty [20,30,35]. 

Endoprosthetic reconstruction, the most common option in limb-salvage surgeries, is an attractive alternative to other surgical options and plays a key role in keeping the patients’ quality of life. This surgical technique can provide early mobilization, stability, and weight-bearing for patients [12,36], and has been reported to result in a better and more predictable outcome than allograft reconstruction [37]. 

On the other hand, the disadvantages of endoprosthesis surgery have to be considered, such as infection, loosening of prosthesis, joint stiffness, limb-shortening or lengthening and implant fracture. In particular, infection associated with endoprosthesis is a serious problem among these complications. Infections at the surgical site were observed in 11% of adult patients with bone tumors treated with endoprosthetic reconstruction [38], while the infection rate in children has been reported to be about 20% [39]. Once an infection has occurred, urgent treatment must be conducted with both intensive debridement and optimal antibiotic therapy during the acute phase. Furthermore, a two-stage revision surgery, with about 70% success rate, should be performed in the chronic phase. In some cases, an amputation will ultimately be necessary [40].

Autografts are commonly performed using vascularized fibula grafts to fill diaphyseal bone defects after wide tumor resection. The fibula is more suitable for the reconstruction of the upper limbs than that of the lower limbs; because the fibula alone does not have enough strength to sustain the body weight, leading to a high risk of fractures. Thus, in the case of lower limb reconstruction, the vascularized fibula is normally inserted into the allograft such as the tibia in order to reinforce its total bone grafting [41]. This combination grafting has shown excellent results in bone union and limb function [42]. Furthermore, tumor-bearing bones have been used successfully as another option for autografts [43,44]. First, the tumor-bearing bone is removed and sterilized by extracorporeal irradiation [43] or pasteurization [44]. Then, this sterilized bone is re-implanted back into place to replace the bone defect.

Allografts from a bone bank can be used for the reconstruction of bone defects after limb-salvage resection of malignant bone tumors with satisfactory long-term results [45,46]. Allograft reconstruction in children has several advantages such as biological integration and joint preservation compared with metal implants. However, its complications include infection [46], fracture [47], and nonunion of the host-allograft junction [48]. These complications normally occur within the first three years after surgery [49].

Rotationplasty was designed for the reconstruction of bone defects around the knee following above-knee amputation. Currently, this surgical technique is recognized as an option standing between amputation and limb-salvage surgery. Rotationplasty can be usually applied to tumors located in the femur or proximal tibia especially in patients with remaining growth potential. First, the distal femur with the tumor is removed; the distal part of lower leg and ankle are preserved. Second, the tibia and foot are rotated 180° and attached to the femoral stump. Consequently, the ankle is at the appropriate height of the contralateral knee [24], playing a role as a functional “knee” joint, so that the patients are comfortable with below knee artificial prosthesis [50]. The major disadvantage is the cosmetic problem, which can be unacceptable especially to adolescents and females [51]. Informed consent based on sufficient discussion with the patient and the patients’ family should be required.

#### 2.1.4. Local Recurrence after Surgery

Although there is no difference in disease-free survival between limb-salvage surgery and amputation in patients with high-grade osteosarcoma, limb-salvage surgery increases the risk of local recurrence [21,23]. Local recurrence is directly correlated to the surgical margins and the degree of tumor necrosis following chemotherapy. Picci *et al*. reported that local recurrence rate in a limb-salvage or amputation surgery group was 7% or 2.4%, respectively, in patients with high-grade osteosarcoma [52]. Bacci *et al*. reported that local relapse occurred in 5% of surgically-treated patients with osteosarcoma who had an inadequate surgical margin [53]. In this study, amputation/disarticulation or 2nd limb-salvage surgery with chemotherapy was conducted in the local recurrence group.

#### 2.1.5. Lung Metastasectomy

Patients with metastatic osteosarcoma at diagnosis, commonly located in the lung, have a poor prognosis. Several papers have demonstrated that the overall survival of patients with metastatic osteosarcoma ranges from 10–50% [7,54,55], depending on the localization and the number of metastatic foci [56,57]. Lung metastasectomy has been shown to increase or prolong survival in osteosarcoma patients with lung metastasis [58,59,60], and surgical resection plays an important role in the management of such patients. In fact, the 5-year survival of patients with complete lung metastasectomy was 12–23%, whereas that of patients without aggressive surgical resection was 2.6% [61,62]. Almost all of those patients will die of their disease without aggressive resection. Therefore, complete lung metastasectomy should be performed to prolong survival of the patients whenever possible.

### 2.2. Chemotherapy

#### 2.2.1. Standard Chemotherapy

Chemotherapy is the most common treatment for patients with osteosarcoma since the 1970s. Since chemotherapy or surgery alone is not effective enough for osteosarcoma treatment, a combination of them is usually applied [11]. The current standard regimens consist of neoadjuvant and adjuvant chemotherapy. Neoadjuvant chemotherapy, introduced in 1978 [14], can induce tumor necrosis in the primary tumor, facilitate surgical resection, and eradicate micrometastases [31]. 

Tumor necrosis is usually assessed as follows: Grade I (no necrosis), II (50% to 95%), III (more than 95% but less than 100%), and IV (100%) [14]. Patients with tumor necrosis of more than 90% are classified as good responders to chemotherapy, whereas those with less than 90% as poor responders. The degree of tumor necrosis following neoadjuvant chemotherapy, known to be a prognostic marker, is useful to verify the effectiveness of the chemotherapy treatment [27,63]. Moreover, the survival rate is usually estimated by the histologic response of the tumor to the neoadjuvant chemotherapy at the time of surgical resection [53,64]. Thus, drugs for adjuvant chemotherapy should be selected based on the degree of tumor necrosis induced by neoadjuvant chemotherapy. The current standard protocol for multi-agent chemotherapy (MAP) consists of doxorubicin, cisplatin and high-dose methotrexate (MTX) with leukovorin-rescue, ±ifosfamide [65] (Table 3), which provides approximately 70% overall survival for patients with primary osteosarcoma [66]. Indeed, doxorubicin and methotrexate have been applied as successful chemotherapy agents for patients with localized osteosarcoma [67,68,69,70]. Moreover, the addition of cisplatin and ifosfamide to doxorubicin and MTX has significantly improved the clinical results of osteosarcoma treatment [71,72]. A meta-analysis has demonstrated that multi-agent regimens including MAP ± ifosfamide have significant better outcomes than 2-drug regimens, whereas there is no significant difference between MAP and MAP with ifosfamide [73]. By contrast, other agents such as vincristine, bleomycin, and dactinomycin were reported to be ineffective [74,75].

**Table 3:** Standard chemotherapeutic agents in osteosarcoma [31].

Agent	Mechanism of action
**Doxorubicin**	Doxorubicin intercalates at point of local uncoiling of the DNA double helix and inhibits the synthesis of DNA and RNA.
**Cisplatin**	Cisplatin, binding to tumor DNA, inhibits the DNA synthesis via the DNA crosslinks.
**Methotrexate**	Methotrexate is a folate antimetabolite and inhibits the synthesis of purine and thymidylic acid by binding dihydrofolate reductase.
**Ifosfamide**	Ifosfamide causes crosslinking of DNA strands, which inhibits the synthesis of DNA and protein.

#### 2.2.2. Novel Chemotherapies

Pemetrexed (PMX), a new-generation antifolate-drug that targets multiple enzymes in folate metabolism, has a wider range of action than MTX [76]. PMX is currently approved as first or second line treatment alone or in combination with cisplatin for malignant tumors [77,78,79]. However, even though PMX was less toxic, it did not induce apoptosis more effectively than MTX in OS cell lines *in vitro* [80]. In the same vein, a phase II study of PMX conducted in patients with high-grade, advanced, or refractory osteosarcoma to assess response rate and toxicity (NCT00523419) showed that PMX was well-tolerated but did not enhance anti-tumor activity [81,82].

In another approach, liposomal muramyl tripeptide phosphatidyl ethanolamine (L-MTP-PE) is the first new agent approved for the treatment of nonmetastatic osteosarcoma in the last 30 years. This agent is a potent stimulator of macrophages and monocytes, and induces the secretion of several cytokines including interleukin-1, -6, and tumor necrosis factor-α. A recent randomized trial demonstrated that L-MTP-PE with MAP and ifosfamide significantly improved the 6-year overall survival of patients with primary osteosarcoma from 70% to 78% [83]. Therefore, the combination of L-MTP-PE and MAP with ifosfamide is strongly expected to become a “routine” agent for the treatment of patients with osteosarcoma [84].

#### 2.2.3. Metastatic Osteosarcoma

Patients with metastatic osteosarcoma at initial diagnosis still show a poor prognosis, basically related to the number and localization of the metastases. The treatment for metastatic osteosarcoma patients with a poor response to the standard chemotherapy remains an unsolved problem. The overall survival rate of these patients ranges from 10% to 50% [7,54,55], while that of patients with localized osteosarcoma is around 60% to 70% [27,85,86]. So far, studies with the multi-agent chemotherapy MAP ± ifosfamide have shown to be effective for patients with osteosarcoma and have resulted in 2-year progression-free survival rates of 39% and 58% for patients with lung and bone metastatic osteosarcoma, respectively [56]; however, a long-term follow-up is needed. Under the present circumstances, there is no satisfactory alternative to vigorous multi-agent chemotherapy including new agents, primary tumor control, and complete metastasectomy for the treatment of metastatic osteosarcoma [87].

### 2.3. Radiotherapy

#### 2.3.1. Photon and Proton/Carbon Ion Radiotherapy

Currently, conventional photon radiotherapy at a dose of 50–60 gray (Gy) plays only a minor role in the multi-disciplinary approach involving surgery and chemotherapy to maximize tumor control [88,89]. Since osteosarcoma is a relatively radio-resistant tumor, the use of radiotherapy is limited in the treatment of primary osteosarcoma and is usually not applied as a first-choice [90]. Thus, photon radiotherapy is commonly applied only in patients with unresectable or inaccessible osteosarcoma occurring in axial sites including the head, neck, spine, and pelvis as a palliative option [88,91]. Conventional radiotherapy in combination with surgery and chemotherapy has been reported to improve long-term survival in osteosarcoma of the spine or the pelvis [92,93]. Irradiation has been conducted to suppress tumor viability and decrease the local relapse as a neoadjuvant, adjuvant setting [94,95].

Recent reports have also demonstrated the efficacy of heavy ion radiotherapy for patients with osteosarcoma [96,97]. Heavy ion particles including carbon ions or protons offer a higher physical selectivity and biological effectiveness compared to photon radiotherapy [98]. Kamada *et al*. demonstrated 3-year local control and overall survival rates of 73% and 45%, respectively, in patients with bone and soft tissue sarcomas treated with carbon ion radiotherapy, including 15 patients with unresectable osteosarcoma of the pelvis (10 patients) or the spine (5 patients). The total dose ranged from 52.8 to 73.6 Gy [99]. Proton therapy was conducted in a total of 55 patients with unresectable or partially-resected osteosarcoma in a study carried out at Massachusetts General Hospital [96]. This study demonstrated that the local control (LC) rates at 3 and 5 years were 82 and 67%, respectively. The mean dose was 68.4 Gy. Moreover, the 5-year LC and overall survival rates for 78 patients with unresectable osteosarcoma of the trunk were 61% and 32%, respectively [97]. Thus, carbon ion/proton radiotherapy in combination with a standard therapy can offer better local control for patients with unresectable or partially-resected osteosarcoma.

On the other hand, extracorporeal irradiation (ECI), first described in 1968 [100], has been used in reimplantation of the tumor-bearing bone as an innovative technique [100,101,102,103]. This biological limb reconstruction normally consists of en bloc tumor resection, extracorporeal irradiation of the resected pathological bone, and reimplantation of the irradiated bone. The use of irradiated bone can provide the anatomical precision of the reimplanted bone segment, avoid immunological rejection, and reduce the risk of postoperative infection [100,101,102,103].

#### 2.3.2. Prophylactic Irradiation for Lung Metastasis

Prophylactic irradiation for patients with lung metastatic osteosarcoma was reported as a postoperative treatment in the late 1970s [104,105,106,107]. Most of the previous studies reported no benefit with prophylactic pulmonary irradiation for the patients [104,105,108,109]; whereas only one study has demonstrated an overall survival of 66% in 41 patients with osteosarcoma treated with both chemotherapy and 20 Gy of prophylactic lung irradiation [110]. In addition, there are serious complications with prophylactic lung irradiation including downregulated respiratory function and opportunistic infections due to the decreased immunocompetence [110]. Taken together, prophylactic lung irradiation has not demonstrated a clear benefit to date.

#### 2.3.3. Palliative Radiotherapy for Painful Bone Metastases

Radiation therapy can be used as an effective, palliative option for painful bone metastases. Samarium-153 ethylene diamine tetramethylene phosphonate (153Sm-EDTMP) is a bone-seeking radiopharmaceutical for palliation of bone metastases introduced in 1998 used at a standard palliative dose of 1 mCi/kg [111]. This therapeutic option has demonstrated palliative benefit for patients with bone metastatic osteosarcoma [112,113]. In the case of high-dose treatment with 153Sm-EDTMP (maximum 30 mCi/kg) autologous stem cell rescue is necessary to avoid myeloablation. Lower doses (1 mCi/kg) of 153Sm-EDTMP, combined with bisphosphonates, chemotherapy ± radiation may provide better palliation of bone metastases in osteosarcoma [111]. In summary, 153Sm-EDTMP may be considered as a palliative therapy in bone metastatic osteosarcoma, although further evaluations in controlled clinical trials are needed to elucidate its benefit [111,114].

## 3. Emerging Therapies and New Therapeutic Targets

New strategies and innovative therapeutic approaches are needed to further improve survival in patients with osteosarcoma. The understanding of the tumor microenvironment and identification of new potential targets will lead to a next-generation standard therapy for osteosarcoma treatment. At present, several new therapeutic agents targeting signal transduction pathways of the bone metabolism or the immune system, as well as novel drug delivery systems have been evaluated or are currently undergoing evaluation for efficiency and toxicity in clinical trials.

### 3.1. Immunomodulation

#### 3.1.1. Interferons (IFNs)

Interferons (IFNs) are a group of cytokines with pleiotropic effects such as immunostimulation, antiangiogenic activity and direct antitumor effect [115], and their efficacy in osteosarcoma has been shown *in vitro* and *in vivo* [116,117,118,119]. However, little has been shown on the efficacy of IFNs in osteosarcoma in patients. A Scandinavian study showed a 10-year metastases-free survival rate of 43% in patients with high-grade osteosarcoma that had been treated with IFN-α for 3–5 years [120]. In addition, the 5-year disease-free survival was 63% in a pilot study of patients with non-metastatic osteosarcoma treated with IFN-α for 3–5 years [121]. On the other hand, there were no advantages between IFN-β and standard chemotherapy in a German study with localized osteosarcoma [122]. Currently, the efficacy of IFN-α in high-grade osteosarcoma is being investigated by the international collaboration EURAMOS-1 and these results remain to be published.

#### 3.1.2. Granulocyte-Macrophage Colony-Stimulating Factor (GM-CSF)

Granulocyte-macrophage colony-stimulating factor (GM-CSF) is another immunomodulatory cytokine that has been tested in osteosarcoma. In a phase I study, aerosolized GM-CSF was shown to be feasible, safe, and effective in some patients with melanoma or Ewing sarcoma [123]. Furthermore, GM-CSF was well-tolerated in a phase II trial of patients with first isolated pulmonary recurrence of osteosarcoma. By contrast, GM-CSF had no immunostimulatory effects on the tumor relapse and lung metastases [124].

### 3.2. Intracellular Signal Pathway

#### 3.2.1. Src

Src is a tyrosine kinase involved in osteoclast activity. Src activates tumor cell-motility and invasion [125]. Small molecule therapy with inhibition of the Src kinase pathway can induce anti-proliferative and pro-apoptotic activity in osteosarcoma cell lines and xenograft models [126,127]. Thus, Src may be a promising therapeutic target for the treatment of osteosarcoma. A tyrosine kinase inhibitor (dasatinib) has been shown to suppress the function of Src *in vitro*. However, dasatinib has shown no effect on tumor growth and metastasis in osteosarcoma *in vivo* [128]. At present, the Src tyrosine kinase inhibitor AZD0530 (saracatinib), a Src inhibitor for c-Src and v-Abl, is being investigated in a phase II clinical trial in lung metastatic osteosarcoma with complete metastasectomy by the Sarcoma Alliance Research through the Collaboration Global Cooperative Network (SARC012, NCT00752206). 

#### 3.2.2. Mammalian Target of Rapamycin (mTOR)

The mammalian target of rapamycin (mTOR), a serine-threonine protein kinase, induces the progression from the G1 to the S phase of the cell cycle [129]. This protein kinase is involved in the regulation of protein synthesis and its signal pathway is considered an attractive therapeutic target for the treatment of tumors including osteosarcoma [130]. Indeed, inhibition of the mTOR pathway has been shown to be effective in a model of murine osteosarcoma [129,131]. Furthermore, rapamycin monotherapy has been shown to be effective on osteosarcoma and several other human tumor xenograft models *in vivo* [132]. The mTOR inhibitor ridaforolimus has been studied in a phase II trial of patients with advanced bone and soft tissue sarcomas. This study showed that single-agent ridaforolimus improved progression-free survival in advanced sarcomas including osteosarcoma [133]. Currently, there are several ongoing clinical trials using mTOR inhibitors in patients with osteosarcoma. The results from these studies may provide the useful insight concerning the potential therapeutic value of mTOR inhibitors in osteosarcoma treatment. 

#### 3.2.3. Hedgehog (Hh)

The Hedgehog (Hh) signaling pathway regulates multiple processes including cell proliferation and differentiation during embryonic and postnatal development [134]. There are three different Hh pathway ligands in mammals, Sonic Hh, Indian Hh, and Desert Hh, which bind to the transmembrane receptor patched (PTCH) to drive the Hh signaling pathway. PTCH normally inhibits Smoothened (SMO) activation in the inactive state [135]. Hh ligands binding to PTCH induce SMO activation and downstream signaling of the Hh pathway. Recently, aberrant activation of the Hh pathway has been reported in various cancers [136]. Several Hh inhibitors have shown potential as anti-cancer agents in *in vivo* experiments [137,138] and some clinical trials [139,140,141]. Inhibition of Hh signaling has also been shown to down-regulate cell proliferation in murine osteosarcoma cells [142]. Therefore, Hh inhibitors, which are effective in SMO inactivation, may be considered as novel therapeutic agents in osteosarcoma.

### 3.3. Tyrosine Kinase Receptors

#### 3.3.1. Insulin-like Growth Factor Receptor (IGF-R)

The insulin-like growth factor receptor (IGF-R) is a membrane tyrosine kinase receptor. There are two subtypes of IGFR: IGF-1R and IGF-2R. IGF-R expression at the mRNA level has been detected in osteosarcoma cell lines: in particular, IGF-2R was overexpressed in all tested osteosarcoma cell lines [143,144]. Increase of IGF-1R expression is correlated with tumor metastases and overall survival in patients with osteosarcoma [145]. 

There are several human monoclonal antibodies (mAbs) against IGF-1R available, and some of them have been or are currently being investigated in phase I/II clinical trials including patients with osteosarcoma. In a phase I study with the monoclonal antibody RG1507, an IGF-1R antagonist, patients with osteosarcoma showed a positive response to treatment with the antibody [146]. Moreover, a phase II trial with RG1507 (NCT00615680) targeting IGF-R in osteosarcoma and other sarcomas is still ongoing [147]. The human mAb SCH 717454 (robatumumab) was not effective *in vitro* but significantly increased event-free survival *in vivo* in several models with solid tumors including osteosarcoma [148]. A phase II study with robatumumab is ongoing for patients with relapsed osteosarcoma or Ewing’s sarcoma (NCT00617890). Cixutumumab is a human mAb targeting membrane-bound IGF-1R. This agent inhibits IGF-1 and its downstream signaling. A Phase I study (NCT00609141) of cixutumumab for young patients with relapsed/refractory osteosarcoma and other solid tumors aimed to determine the optimal dose, toxicity, and pharmacokinetics has been completed recently but has not been published yet. In addition, cixutumumab is administered to patients with relapsed or refractory solid tumors including osteosarcoma to determine its response rate and side effects such as toxicity in an ongoing phase II study (NCT00831844). 

#### 3.3.2. Human Epidermal Growth Factor Receptor 2 (HER-2)

Human epidermal growth factor receptor 2 (HER-2) was highly expressed in about 40% of osteosarcoma patient samples [149]. Increased expression of HER-2 was found to occur more frequently in patients who presented with metastases at the time of diagnosis and at relapse. Expression of HER-2 also correlated with worse tumor necrosis grade at the time of resection and with a worse event-free survival [149]. For patients with cytoplasmic HER-2 expression, no association of high HER-2 expression with the response to preoperative chemotherapy or prognosis has been found [150]. A phase II trial (NCT00023998) of trastuzumab, a mAb targeting HER-2, was initiated in patients with metastatic osteosarcoma from 2001 to 2010. Trastuzumab was administered together with conventional chemotherapy. This study demonstrated that there was no significant difference in event-free and overall survival between the HER-2-positive group treated with trastuzumab and the HER-2-negative group treated with cytotoxic chemotherapy alone [151].

#### 3.3.3. Vascular Endothelial Growth Factor (VEGF)

Vascular endothelial growth factor (VEGF) expression has been reported to be involved in pulmonary metastasis development and decrease of both overall and event-free survival in patients with osteosarcoma. In addition, VEGF receptor (VEGFR) has been shown to be overexpressed in osteosarcoma cell lines [143]. In particular, VEGFR-3 has been shown to be inversely correlated with both overall and event-free survival [144]. A phase II trial of sorafenib, a VEGF inhibitor, has been conducted in patients with relapsed and unresectable high-grade osteosarcoma. The progression-free survival was 4 months, the overall response and disease control rates were 14% and 49%, respectively [152].

#### 3.3.4. Platelet-Derived Growth Factor (PDGF)

Platelet-derived growth factor (PDGF) and its receptor (PDGFR) are known to activate cell proliferation and differentiation of both osteoblasts and osteoclasts [153]. In osteosarcoma, PDGF/PDGFR signaling is correlated with tumor growth and progression [154]. At present, there are two contradictory reports about the correlation between PDGF and tumor prognosis in osteosarcoma [155,156]. Imatinib mesylate (STI-571), a tyrosine kinase inhibitor, has been considered to be a possible target for a novel treatment of osteosarcoma [157]. However, imatinib has shown little efficacy on patients with refractory osteosarcoma in a phase II study (NCT00031915) [158].

### 3.4. Drug Delivery System

Modification in the delivery system of current chemotherapy agents may result in clinical benefits. Liposomal encapsulation of doxorubicin has been shown to be effective *in vitro* and *in vivo* [159,160]. Furthermore, the efficacy of SLIT cisplatin has been investigated, in particular in osteosarcoma patients [67]. The sustained release lipid inhalation targeting (SLIT) cisplatin can provide a prolonged therapeutic effect of cisplatin in the lung. A Phase I/II clinical trial with SLIT cisplatin (NCT00102531) was initiated in 2005 in patients with relapsed/progressive osteosarcoma metastatic to the lung. Early results show promising activity and suggest that SLIT cisplatin is well tolerated; however, final results remain to be published [161]. In addition, a phase II clinical trial (NCT01650090) using SLIT cisplatin in osteosarcoma patients with complete surgical resection following one or two prior pulmonary relapses was initiated in 2012. The results of these studies will provide new insights into the clinical benefits of this therapeutic approach.

### 3.5. Bone Metabolism

#### 3.5.1. Bisphosphonates

Bisphosphonates, which inhibit osteoclast-mediated bone resorption, have been shown to suppress pulmonary metastasis and improve overall survival in an osteosarcoma model *in vivo* [162]. Several different types of bisphosphonates have been studied *in vitro* and in osteosarcoma models *in vivo* [163,164]. For example, Alendronate inhibits cell invasion and induces cell apoptosis in human osteosarcoma cell lines [165]. In turn, there are conflicting results about the efficacy of zoledronate, which is a third-generation bisphosphonate, in osteosarcoma models *in vivo*. Zoledronate downregulated tumor growth and lung metastasis in some models with osteosarcoma [166,167], whereas in others, it inhibited neither primary tumor growth nor did it reduce lung metastasis in spite of inhibiting osteolysis and tumor-induced bone formation [168,169].

Recently, two clinical trials with zoledronate for osteosarcoma treatment have been initiated. A phase I/II trial (NCT00742924) of zoledronate, together with conventional chemotherapy including ifosfamide and etoposide, has recently been completed in patients with newly-diagnosed metastatic osteosarcoma. The aim of this study was to determine the optimal dose of zoledronate to minimize side-effects. The results remain to be published. In addition, a phase II/III trial (NCT00691236) of zoledronate used as a single agent or together with adjuvant chemotherapy in high-grade osteosarcoma is ongoing. The response to zoledronate and disease-free survival will be evaluated.

#### 3.5.2. RANK/RANKL/OPG Axis

Osteosarcoma is strongly associated with a deregulated balance of the molecular triad receptor activator of nuclear factor-κβ (RANK), its ligand (RANKL) and the decoy receptor osteoprotegerin (OPG), leading to pathological bone remodeling and metabolism [170,171]. In a recent study, functional RANK expression has been reported in several human osteosarcoma cell lines [172]. Furthermore, RANKL has been shown to be expressed in patients with osteosarcoma, and the 5-year event-free survival was poor (less than 20%) in patients with high RANKL expression [173]. Moreover, OPG indirectly induced both the prevention of tumor-induced osteolysis and the inhibition of tumor-associated development that improved overall survival rate *in* osteosarcoma *in vivo* [174]. In addition, inhibition of RANK in osteosarcoma cell lines has been demonstrated to reduce cell motility and invasion [175]. Thus, the RANK/RANKL/OPG axis possibly plays a role in the development of osteosarcoma, and may be a novel therapeutic target in RANK-positive osteosarcoma.

Denosumab, which inhibits bone resorption by osteoclasts, is a humanized monoclonal antibody to RANKL for the treatment of osteoporosis and bone metastasis [176,177]. There is currently no ongoing clinical trial with denosumab for the treatment of osteosarcoma. However, the efficacy of denosumab has been demonstrated in clinical trials of giant cell tumor of bone [178]. Indeed, a recent study showed that denosumab was able to reduce tumor size by more than 90% in all patients with RANK-positive giant cell tumors [179]. A phase II trial of denosumab (NCT00680992) is currently being conducted in patients with recurrent or unresectable giant cell tumor of bone. These results may become a trigger for the development of innovative treatments for patients suffering from osteosarcoma.

## 4. Future Perspectives

The prognosis of localized osteosarcoma has dramatically improved in the second half of the last century with the introduction of multi-modal treatments including vigorous surgery, multi-agent chemotherapy, and radiation therapy. However, survival rates have not improved after that. Furthermore, the prognosis of metastatic and recurrent osteosarcoma still remains poor. Thus, novel therapeutic agents are clearly required, and various clinical studies with new agents have been conducted recently or are ongoing. However, they have not been successful yet in further improving survival rates for patients with localized or metastatic osteosarcoma. 

One reason for this is that new drugs are usually tested in patients with refractory osteosarcoma such as metastatic or recurrent disease or osteosarcoma resistant to standard treatment. Also, the number of patients with osteosarcoma is small due to its rarity. Consequently, clinical trials evaluating the potential of innovative therapies compared to conventional options take long time periods before significant results can be obtained. Therefore, large multicenter and international collaborations are required in order to facilitate the development of innovative drugs and therapeutic approaches. Another challenge is that osteosarcoma is a heterogeneous and chaotic tumor with a variety of genetic behaviors and clinical features, resulting in a prognosis variability between individuals [180]. Thus, the optimal therapeutic approach should be more precisely defined for each subgroup of osteosarcoma populations with a precise stratification of the patients. Further investigations are required to establish a novel standard treatment for patients with osteosarcoma. 
